# The Challenges of Eradicating Pediatric *Helicobacter pylori* Infection in the Era of Probiotics

**DOI:** 10.3390/children9060795

**Published:** 2022-05-28

**Authors:** Lorena Elena Meliț, Cristina Oana Mărginean, Maria Oana Săsăran

**Affiliations:** 1 Department of Pediatrics I, George Emil Palade University of Medicine, Pharmacy, Science, and Technology of Târgu Mureș, Gheorghe Marinescu Street No. 38, 540136 Târgu Mureș, Romania; lory_chimista89@yahoo.com; 2 Department of Pediatrics III, George Emil Palade University of Medicine, Pharmacy, Science, and Technology of Târgu Mureș, Gheorghe Marinescu Street No. 38, 540136 Târgu Mureș, Romania; oanam93@yahoo.com

**Keywords:** *Helicobacter pylori* infection, probiotics, children

## Abstract

*Helicobacter pylori* (*H. pylori*), the most common infection of childhood, results in life-threatening complications during adulthood if left untreated. Most of these complications are related to *H. pylori*-induced chronic inflammation. The dysbiosis caused by *H. pylori* is not limited to the gastric microenvironment, but it affects the entire gastrointestinal tract. Eradication of *H. pylori* has recently become a real challenge for clinicians due to both the persistent increase in antibiotic resistance worldwide and the wide spectrum of side effects associated with the eradication regimens resulting; therefore, there is an urgent need for more effective and less noxious treatment options. Thus, probiotics might be a promising choice in both adults and children with *H. pylori* infection since their role in improving the eradication rate of this infection has been proved in multiple studies. The positive effects of probiotics might be explained by their abilities to produce antimicrobial compounds and antioxidants, alter local gastric pH, and subsequently decrease *H. pylori* colonization and adherence to gastric epithelial cells. Nevertheless, if used alone probiotics do not considerably increase the eradication rate.

## 1. Introduction

*Helicobacter pylori* (*H. pylori*), the most common bacterial infection worldwide might be defined as the bacterium of childhood since it is usually acquired during this period of life and it might result in life-threatening complications during adulthood including peptic ulcers, adenocarcinoma, or gastric lymphoma [1,2]. Moreover, several complications of this infection were also reported in children, among which include growth retardation, idiopathic thrombocytopenic purpura, and vitamin B12 or iron deficiency anemia [3,4,5]. Although this infection affects more than 50% of the population worldwide, its prevalence reaches up to 80% in developing countries [6]. Taking into account that without treatment, *H. pylori* infection leads to chronic inflammation of the gastric mucosa, it is not surprising that most of the above-mentioned complications seem to be related to this local chronic inflammatory process [7,8]. In addition, a Japanese study proved that the risk of developing gastric ulcer or cancer is directly related to the age of the first infection underlining that in the setting of early life infection, the risk is considerably higher [9]. Therefore, the proper monitoring and effective treatment of this infection in children definitely represents the cornerstone of prevention in terms of gastric *H. pylori*-associated carcinogenesis during adulthood. 

According to the World Health Organization, *H. pylori* is defined as a class I carcinogen based on the fact that gastric cancer originating from this infection represents one of the most common cancer-related causes of death worldwide [10]. Three major factors were identified as the main contributors to gastric carcinogenesis proving that the complexity of this process is not entirely related to *H. pylori* virulence constituents, but also to the host’s genetic background and to the general and local environmental factors [11]. Innate immunity via toll-like receptors (TLRs) represents a major component of host defense mechanisms against the progression of this infection into a chronic inflammation of the gastric mucosa [12]. Thus, these TLRs own a dichotomous role since they might either promote or suppress the progression of this infection by enabling different host-specific or bacterial-related mechanisms depending on the gastric microenvironment’s composition [13]. 

The other side of the carcinogenesis process is represented by the escaping mechanisms that *H. pylori* developed over time in order to survive the host’s gastric microenvironment and to escape all the aforementioned host’s defense immune response, enabling its long-term persistence in the host’s stomach. These complex survival mechanisms comprise the inhibition of cathepsin X, the ability to alter the structure of its lipopolysaccharides, and to shape T-cell responses, all contributing to *H. pylori*’s resistance against the most commonly used drugs for its eradication [14]. 

Unsurprisingly, several studies pointed out that the bacterial resistance rates are continuously increasing, reaching up to 17.5% for clarithromycin and 34.9% for metronidazole [15]. Moreover, *H. pylori* resistant strains to levofloxacin, which in fact is considered a rescue therapy, have been reported [15] underlining the urgent need to design and implement more complex targeted eradication regimens based on more than the susceptibility of this bacterium to antibiotics, by also involving the host’s immune components and targeting the promotion and augmentation of mechanisms that suppress the transformation of this infection from acute to chronic. 

However, the impact of *H. pylori* infection on gastric microbial diversity, as well as gut microbiome and gut inflammatory parameters, is still far from being elucidated. Animal model and human studies on this topic raised multiple controversies concluding that chronic *H. pylori* infection might result in gut dysbiosis and consequently act as a major contributor to the local and systemic disorders [16]. Thus, we might state that *H. pylori* has the ability to influence bacterial community composition along the entire gastrointestinal tract with subsequent life-threatening long-term consequences in terms of chronic inflammation and further malignant transformation. 

This review aims to assess the challenges in eradicating *H. pylori* infection related to the host’s defense mechanisms, gastric microenvironment, and probiotic usefulness. 

## 2. Gastric Microbiota Diversity and *H. pylori* Infection in Children

The gastric microbiota or gastric microenvironment represents a complex genomic content consisting of microbial communities, the host’s immune system components, and host epithelium [17]. Despite the less hospitable gastric microenvironment, several microorganisms were found to survive these conditions, among which include bacteria or fungi [18]. Thus, regardless of the presence of *H. pylori*, the human stomach is not a sterile environment. Multiple studies performed on adult populations revealed that *Prevotella* and *Streptococcus* account for the majority of the bacterial communities in gastric samples, also identifying *Nesseria*, *Veillonella*, *Haemophilus*, and *Fusobacterium* to be commonly harbored by this microenvironment [18]. In terms of healthy subjects, the information is scarce due to the fact that most studies assessed patients with different *H. pylori*-related or non-*H. pylori* gastropathies, but the most frequently reported bacterial genera among these subjects were *Prevotella*, *Streptococcus*, *Oribacterium*, *Megasphere*, *Propionibacterium*, and *Capnocutophaga* [18,19,20]. When assessing the impact of *H. pylori* infection on the gastric microbial community, studies concluded that this infection decreases both the abundance and diversity of gastric microbiome content [21]. Moreover, *H. pylori*-positive subjects with antral gastritis harbor an increased amount of *Firmicutes* and a decrease in *Proteobacteria* in comparison to those with *H. pylori*-negative gastritis [22]. In terms of atrophic gastritis, studies reported a reduction in *Prevotella* and an increase in *Streptococcus* [23]. 

The complex interplay between *H. pylori*, defensins, and gut microbiota has been revealed by recent research. Defensins belong to the family of antimicrobial peptides, which own an important role in native immune responses to bacteria, viruses, yeast, and fungi and they might represent promising substitutes for antibiotics in order to decrease microbial resistance [24,25]. Thus, defensins, and especially beta-defensins (HBD), regulate the host’s immune responses, being capable to induce important structural changes in the gut microbiome during *H. pylori* infection, but at the same time, their expression is closely influenced by *H. pylori* [26]. This bacterium was proved to exert important activity on a wide spectrum of HBD. Both HBD 1 and 2 were found to be increased in patients with *H. pylori*-positive gastritis in comparison to healthy subjects, but also in the setting of bacterial inflammation indicating that they have a major role against certain injurious stimuli in the stomach [27,28]. In addition, HBD-2 is overexpressed in the corpus of patients with *H. pylori* infection, while HBD-1 is expressed unevenly [29]. Nevertheless, HBD-1 decreased expression was associated with *H. pylori* infection [30], it is documented that this infection has the ability to downregulate HBD-1 expression through NF-κB signaling enabling prolonged survival and persistence in the stomach niche [31]. HBD-3 was found to be mainly expressed in the gut of *H. pylori* individuals, but not in the absence of this infection [32]. Contrariwise, it seems that the release of HBD-3 from *H. pylori* infected cells occurs during the early stage of this infection via a new Epidermal growth factor receptor (EGFR)-activating [33]. HBD-4, otherwise poorly expressed in gastric cells, is mostly up-regulated in gastric inflammation irrespective of the presence of *H. pylori* [26]. Nevertheless, cytotoxin-associated antigen A (cagA) positive *H. pylori* strains were associated with an important increase in HBD-4 expression as compared to cagA negative strains [26]. The findings reported in the literature remain controversial since a recent study indicated that *H. pylori* has the ability to induce only the expression of HBD-2, and not HBD-3 and HBD-4 [25]. 

TLRs represent another crucial component of the host’s immune system. Several studies performed on pediatric subjects with *H. pylori* and non-*H. pylori* gastritis stated a major involvement of these TLRs, especially TLR2, TLR4, and TLR9 in the development of gastric chronic inflammation and their subsequent contribution to triggering systemic inflammation [34,35,36]. The action of these TLRs and their expression is influenced by the composition of the gastric microbial community resulting in a major role of the gastric microbiome in regulating the proper functioning of the host’s innate immune responses. The involvement of the host’s immune system in gastric carcinogenesis goes further through the synthesis of pro-inflammatory cytokines, costimulatory molecules, chemokines, and antigen-presenting molecules as a result of activating the TLR signaling pathways [37]. 

It is a well-documented fact that *H. pylori* infection induces multiple essential mucosal changes in the stomach once it becomes chronic which will further enable the development of a new microbiota involved in gastric carcinogenesis [38]. Therefore, *H. pylori* not only increases the risk of carcinogenesis itself but also promotes the development of a gastric microbiome that supports the malignant transformation of the mucosa. Moreover, a recent review indicated that aside from *H. pylori*, proton pump inhibitors, and bariatric surgery might also influence the composition of the gastric microbial community [39]. Thus, the authors underlined that proton pump inhibitors have the ability to increase gastric microbial diversity. In addition, certain surgical procedures of bariatric surgery were associated with an increase in potentially pathogenic *Proteobacteria* in the gut microbiota; while others were reported to induce a significant decrease in *Clostridiaceae, Lachnospiraceae, Eubacteriaceae, Ruminococcaceae, Carnobacteriaceae, and Coriobacteriaceae* family members along with an enrichment of *Acidaminococcus, Megasphaera, Lactobacillus*, and *Enterobacteriaceae* family members.

The studies performed on pediatric patients that aimed to assess the composition of the gastric microflora remain scarce especially due to the reluctance in performing upper digestive endoscopy in this age group. Nevertheless, the presence of other bacterial communities aside from *H. pylori* represents a major issue regarding gastric carcinogenesis since they were proved to act as a persistent antigenic stimulus or even enter a partnership with *H. pylori* for enabling its persistence and favoring subsequent gastric inflammation [40]. In addition, nitrate-reducing bacteria such as *Staphylococcus epidermidis*, *Micrococcus luteus*, *Staphylococcus haemolyticus*, *Neisseria mucosa, Actinomyces naeslundii,* and *Rothia dentocariosa* were also found to colonize the gastric mucosa increasing once more the risk of gastric carcinogenesis based on their role in enabling the accumulation of N-nitroso compounds and nitrite in this microenvironment [38]. The hypothesis that gastric commensal flora augments *H. pylori*-associated inflammation was also confirmed by animal studies [41]. Moreover, *H. pylori*-positive children were found to have a more diverse and abundant gastric microflora as compared to *H. pylori*-positive adults [42]. A study that included 346 children complaining of dyspeptic symptoms reported 114 patients diagnosed with *H. pylori* infection, also identifying 366 non-*H. pylori* genera among which were 247 Gram-positive and 119 Gram-negative bacteria [43]. Children with *H. pylori* infection harbor also *Neisseria*, *Staphylococcus*, *Streptococcus*, and *Rothia* within their gastric microbial community [43], but *Rothia* was less abundant when compared to infected adults [42]. A lower abundance of *Firmicutes* along with a higher abundance of *Proteobacteria* and *Gammaproteobacteria* were also reported as a major difference between infected pediatric subjects and infected adults [42]. *Haemophilus, Neisseria,* and an unidentified genus of the Neisseriaceae family were also reported to colonize particularly the gastric mucosa of pediatric patients, but not of adult subjects [42]. The impairment of gastric microecology homeostasis occurs during childhood and owns a major contribution in the pathway towards gastric carcinogenesis. 

The diagnosis of *H. pylori* is challenged by the presence of other urease-producing bacteria within the gastric microenvironment aside from *H. pylori*, even in its absence, such as *Pseudomonas plecoglossicida*, *Staphylococcus aureus* and *epidermidis*, *Acinetobacter johnsonii*, *Neisseria flavescens*, *Neisseria mucosa*, *Neisseria meningitidis*, *Neisseria perflava*, *Micrococcus luteus*, and *Rothia mucilaginosa* [43]. An even more challenging fact in terms of diagnosis is represented by the carrier state, especially during childhood, the most common period for acquiring this bacterial infection. In addition, children that carry a low abundance of *H. pylori* within the gastric mucosa are not always deemed to develop gastritis. According to a Chinese study, five out of six subjects that were initially found to be *H. pylori* negative were diagnosed as healthy carriers based on DNA sequencing methods, but with a considerably lower abundance of *H. pylori* ranging between 0.04% and 0.67% [44]. Another study performed on Spanish children concluded that 17 of them carried a minor *H. pylori* abundance, only 0.45% [45]. 

## 3. Antibiotics versus Probiotics in Pediatric *H. pylori* Infection

Eradication of H. pylori has recently become a real challenge for clinicians due to the persistent increase in antibiotic resistance worldwide. The recommended regimen for the eradication of this infection consists of the standard triple therapy involving two antibiotics such as clarithromycin and amoxicillin or metronidazole combined with a proton pump inhibitor [46,47]. This regimen became a standard therapy worldwide during the 1990s based on its high eradiation rates of over 90% [48]. Unfortunately, its efficacy decreased considerably lately to less than 70% due to a wide range of *H. pylori* resistant strains that emerged mainly to clarithromycin, but also to metronidazole and levofloxacin [49,50]. Based on these antibiotic-related concerns, recent studies focus more and more on assessing the role of other potential therapies in the long-term effective eradication of this infection, beginning with the previously proven statement that probiotics might have the ability to contribute to both the eradication of *H. pylori* and the diminishment of therapy-related side effects [51]. 

### 3.1. Antibiotics: Yes or No

Despite the fact that the standard triple regimen was considered the gold standard in the 1990s, multiple studies reported a global resistance to clarithromycin with different resistance rates such as 16% in Japan, 10.6 to 25% in North America, and up to 23.4% in Europe [52,53,54]. The variations regarding resistance rates depend on the policies for antibiotic use in different countries since 49% of clarithromycin resistance was noticed in Spain and only 1% in the Netherlands, indicating that Northern European countries have a stricter policy for antibiotic use as compared to Southern European ones [55]. The resistance rates to metronidazole follow the exact same pattern since they seem to vary between 17% in Europe to 44% in America [56,57]. Moreover, in developing countries, these resistance rates reach up to 100% since metronidazole is used in these areas for treating parasitic and gynecological infections with extremely high incidence [58,59]. Taking into account the aforementioned facts, we might state that there is a crucial need for developing a more effective treatment for the proper eradication of this infection, especially during childhood. 

Among the recently recommended eradication strategies, we recall bismuth quadruple therapy used for 14 days which consists of tetracycline, metronidazole, bismuth, and a proton pump inhibitor [60]. The implementation of this regimen was meant to be a first-line eradication treatment for the countries that reported a high incidence of clarithromycin resistance, and as a second-line therapy in cases with a failure of classical triple therapy eradication [46,47]. The issue related to metronidazole resistance was solved by using prolonged high doses during this regimen [61]. Nevertheless, this regimen is limited mainly by the poor availability of bismuth salts and tetracycline in certain countries. In addition, according to several meta-analyses, the efficacy of bismuth quadruple therapy seems to be similar to that of clarithromycin-standard triple therapy [62] (Table 1). 

A similar therapy, but without bismuth, involves a proton pump inhibitor along with all three antibiotics, clarithromycin, metronidazole, and amoxicillin administered for 10 to 14 days, also known as non-bismuth quadruple concomitant therapy, but it is limited by the increased number of pills that must be taken [46,47]. In spite of the potential reduction in its efficacy that might be caused by clarithromycin, this regimen proved considerably higher eradication rates when compared to standard triple therapy [63] (Table 1). 

Sequential therapy involves the same antibiotics as in standard triple therapy, but these antibiotics are administered for a period of 5 days each associated with a proton pump inhibitor during the entire treatment length. Thus, sequential therapy supposes 5 initial days of amoxicillin, which will be associated with clarithromycin and metronidazole for the following 5 days [5]. In terms of efficacy, a recent meta-analysis pointed out that sequential therapy has an eradication rate of 84.1%, while standard triple therapy was effective in 75.1% of the cases [64]. Nevertheless, the efficacy of sequential therapy increases in the setting of single clarithromycin-resistant strains accounting for an 80.9% eradication rate in comparison to only 40.7% when standard triple therapy was used [64] (Table 1). 

Hybrid therapy is based on the hypothesis that amoxicillin has the ability to disrupt the bacterial cell wall and it should be used alone initially in the eradication of *H. pylori* in order to prevent the transfer of the antibiotic outside the bacterial cell through efflux channels [65]. Therefore, this therapy recommends 7 days of proton pump inhibitor combined with amoxicillin followed by another 7 days of quadruple therapy consisting of three antibiotics: amoxicillin, metronidazole, and clarithromycin in association with a proton pump inhibitor. The scarce evidence regarding the use of this therapy does not reveal a considerable higher efficacy of hybrid therapy in comparison to sequential therapy [46,47,66] (Table 1). 

Further studies implemented the use of levofloxacin instead of clarithromycin in triple or sequential therapies with an eradication rate of over 90% in areas with a low local levofloxacin resistant rate [62]. Although less common, the resistance rates to quinolones range from 20% in Europe to 15% in America and 10% in Asia due to their frequent use in urinary tract infections [67]. Levofloxacin-based therapies are usually recommended only as second-line regimens when clarithromycin and/or metronidazole-based therapies fail in eradicate *H. pylori* [68] (Table 1).

**Table 1:** Antibiotics in *H. pylori* infection—pros and cons.

Therapeutic Regimens	Description	Strengths	Limitations
Standard triple regimen	2 antibiotics—clarithromycin and amoxicillin or metronidazole + PPI or [46,47]	recommended regimen/gold standard [46,47] eradication rate > 90% in the 1990s [48]	efficacy ↓ < 70% => *H. pylori* resistant strains to clarithromycin, metronidazole or levofloxacin [49,50]. resistance rate to clarithromycin 49% in Spain [55]. resistance rates to metronidazole 17% in Europe to 44% in America [56,57], up to 100% in developing countries [58,59]
Bismuth quadruple therapy	tetracycline, metronidazole, bismuth, and IPP for 14 days [60]	first-line eradication treatment for countries with a high incidence of clarithromycin resistance [46,47] second-line therapy if classical triple therapy eradication fails [46,47] metronidazole resistance → solved using prolonged high doses during this regimen (Lee et al., 2015) [61] efficacy of bismuth quadruple therapy seems to be similar to that of clarithromycin-standard triple therapy [62]	poor availability of bismuth salts and tetracycline in certain countries [62]
Non-bismuth quadruple concomitant therapy	PPI + all three antibiotics, clarithromycin, metronidazole, and amoxicillin for 10–14 days [46,47]	higher eradication rates when compared to standard triple therapy [63]	increased number of pills that must be take [46,47] potential reduction in its efficacy that might be caused by clarithromycin [63]
Sequential therapy	5 initial days of amoxicillin, followed by clarithromycin and metronidazole for another 5 days, associated with a PPI during the entire treatment length [5]	an eradication rate of 84.1% (Feng et al., 2016) [64] efficacy of sequential therapy increases in the setting of single clarithromycin-resistant strains accounting for an 80.9% eradication rate [64]	resistance rates to clarithromycin
Hybrid therapy	7 days PPI + amoxicillin followed by another 7 days of quadruple therapy consisting of 3 antibiotics, amoxicillin, metronidazole, and clarithromycin + PPI [65]	amoxicillin has the ability to prevent the transfer of the antibiotic outside the bacterial cell through efflux channels [65]	not a considerable higher efficacy in comparison to sequential therapy [46,47,66]
Other regimens	levofloxacin instead of clarithromycin in triple or sequential therapies [62]	eradication rate of over 90% in areas with a low local levofloxacin resistant rate [62] second-line regimens when clarithromycin and/or metronidazole-based therapies fail [68]	resistance rates to quinolones (10–20%) [67]

↓ decrease side effects.

### 3.2. Why Probiotics?

Probiotics represent live microorganisms with beneficial effects on the host’s health when administered in proper amounts [69]. A wide range of microorganisms including both bacteria and fungi are used for probiotics among which include *Lactobacillus*, *Bifidobacterium*, *Saccharomyces*, *Leuconostoc*, *Pediococcus*, *Enterococcus*, *Streptococcus*, *Bacillus*, *Enterococcus*, *Escherichia*, *Clostridium*, *Torulopsis*, etc. [70]. 

In terms of *H. pylori* infection, the most commonly used strains in human or in vivo studies were Lactobacillus rhamnosus GG, Lactobacillus johnsonii La1, Lactobacillus acidophilus, Lactobacillus casei, Lactobacillus gasseri OLL2716, Lactobacillus reuteri, Lactobacillus brevis, Bifidobacterium animalis, Bifidobacterium breve, Bifidobacterium lactis, Propionibacterium freudenreichii, and the probiotic yeast Saccharomyces boulardii [71,72,73]. The effect of probiotics on *H. pylori* infection is complex and although they cannot eradicate the infection if administered alone, they have the ability to increase the eradication rates by up to 10% when used in combination with standard triple or sequential therapies, to diminish the density of *H. pylori* at the level of the gastric mucosa, to improve the patients’ symptoms regardless of the age in comparison to the pretreatment period, and they also reduce the treatment-related side effects such as abdominal distension, diarrhea, or taste disorders [74,75,76,77,78]. In addition, it was also proved that probiotics are effective in improving the histological changes of the gastric mucosa caused by *H. pylori*, indicating that these compounds reduce *H. pylori* density on the luminal part of the gastric epithelium resulting in a considerable long-term improvement of both histological inflammation and disease activity scores in the antrum and corpus [78]. The probiotic’s role in improving the eradication rate of *H. pylori* infection was suggested by multiple studies [79,80,81,82,83], and these effects seem to be related to their abilities to produce antimicrobial compounds and antioxidants, alter local gastric pH, and subsequently decrease *H. pylori* colonization and adherence to gastric epithelial cells [74]. According to McFarland et al., four probiotic mixtures consisting of *L. helveticus*/*L. rhamnosus*, *L. acidophilus*/*B. animalis*, *L. acidophilus*/*E. faecalis*/*B. longum* associated with the eight-strain mixture might result in an eradication rate of more than 90% if used in high doses for approximately 3–5 weeks [83]. Thus, several mechanisms were proposed for explaining the probiotic’s beneficial action in eradicating *H. pylori* infection such as non-immunological and immunological mechanisms, the secretion of antimicrobial substances, the role of competitors for adhesion, and the promotion of mucin secretion [62]. It is a well-documented fact that the mucosal barrier and gastric pH represent the first line of defense against pathogens [62]. The mucosal barrier acts against bacterial invasion through mucin secretion and *H. pylori* was found to suppress certain mucin genes expression such as mucin 1 cell surface associated (MUC1) and mucin 5 cell surface associated (MUC5) in human gastric epithelial cells favoring thus its invasion into the gastric mucosa [84]. Nevertheless, several probiotics among which *L. rhamnosus and L. plantarum* were proved to balance this suppression by increasing the in vitro expression of other two genes involved in gastric mucin producing MUC2 and MUC3, therefore restoring the gastric mucosal permeability and inhibiting the adherence of *H. pylori* to the mucosa [62]. Another potential mechanism involved in supporting *H. pylori* eradication is based on the ability of probiotics to secrete short chain fatty acids such as acetic, lactic, and propionic acids resulting in a reduction of gastric pH [62]. Moreover, certain Lactobacillus spp were found to synthetize proteinaceous toxins with anti-*H. pylori* activity belonging to the class of bacteriocins with different degrees of potence in eradicating this bacterial infection [85]. Probiotic bacteria seem to be involved in inhibiting the adhesion of *H. pylori* to the gastric mucosa by blocking the receptors implicated in this process [86] reinforcing their activity against *H. pylori* mucosal invasion. In terms of immunological mechanisms triggered by probiotics, it was proved that they have the ability to reduce the host’s immunological response to *H. pylori* infection by regulating the anti-inflammatory cytokines secretion resulting in a reduction of local inflammatory response [87] (Table 2). As we already mentioned, beta-defensins are extremely important in host defense mechanism against *H. pylori* infection stenghtenining therefore the innate immune responses. Thus, Schlee et al. stated that probiotics, and especially lactobacilli, might contribute to the up-regulation of certain HBD-2 [88]. 

The aforementioned benefits of probiotics on the gastric mucosa infected with *H. pylori* were proven by multiple studies, especially when a certain mixture of these antibiotics was used [89]. Thus, the use of a five-probiotic mixture including *L. acidophilus*/*B. animalis*, *L. helveticus*/*L. rhamnosus*, *L. acidophilus*/*B. bifidum*, *L. acidophilum*/*E. faecalis*/*B. longum* and the eight-strain mixture associated with standard triple therapy was proved to reduce the antibiotic-associated side effects [89]. The same meta-analysis indicated that antibiotic-triggered diarrhea was considerably reduced when the triple standard eradication regime is administered with a mixture of three multi-strain probiotics, *L. acidophilus*/*B. bifidum*, *L. acidophilus*/*B. animalis* and the eight-strain mixture [89]. A recent review underlined the major influence of *H. pylori* on the bacterial community composition of the entire gastrointestinal tract, which might result in a complex dysbiosis impairing not only the stomach but also especially the gut microbiome [13], proving that children with *H. pylori* infection have a lower amount of fecal *Bifidobacterium spp* and a decreased *Bifidobacterium* to *E. coli* ration in their feces as compared to *H. pylori*-negative children [90]. A more recent study performed on pediatric patients with *H. pylori* infection found a significant decrease of fecal *F. prausnitzii*, an essential bacterium that favors the decrease of gut inflammation triggered by *H. pylori* lipopolysaccharide [16]. The same study suggested that supplementation with *B. lactis* and *L. acidophilus* has a positive effect on *F. prausnitzii* growth. In addition, *H. pylori*-positive children present also a decrease in immunoglobulin A (IgA) levels, which is crucial in regulating gut microbiota [91]. A reduction of gut microbiota fluctuation along with a restriction of the development of antibiotic-resistant bacteria was noticed after probiotics administration in association with standard triple therapies, especially *Bacillus subtilis* and *Streptococcus faecium* [92] (Table 2). 

Multiple studies focused on assessing the role of probiotics in eradicating *H. pylori* infection, either used alone or in combination with standard eradication regimens. Most of these studies underlined the low eradication rate when using a single treatment with probiotics [70]. Thus, the studies performed on healthy positive volunteers indicated a decrease in *H. pylori* colonization and gastric inflammation when using certain species of *Lactobacillus*, suggesting even a decrease in urea breath test after probiotic treatment [93]. Similar results were also encountered when *Bifidobacterium* was administered in *H. pylori*-positive patients with functional dyspepsia [94]. Another study that assessed the role of dietary habits and socio-economic factors in *H. pylori* reinfection pointed out that a low intake of fermented dairies, fruits, and vegetables was significantly lower in re-infected patients [95]. In addition, Rosania et al. highlighted that the use of multi-strain probiotics containing a mixture of *Bifidobacterium* and *Lactobacillus* spp. along with *Streptococcus thermophilus* in adult patients with dyspepsia eradicated the infection in 32.5% of the cases as compared to 0% in the placebo group [96]. The results remain contradictory since a more recent meta-analysis revealed a much lower eradication rate of only 12.4%, suggesting that probiotics have a minor effect on eradicating this infection when used alone [97]. These trends tend to change considerably if probiotics are used in association with the standard eradication therapies. Over the last two decades, most of the studies that assessed the effect of probiotics on *H. pylori* eradication rates suggested that they might improve the eradication outcomes in both adults and children if used combined with standard eradication therapies [70]. Thus, a recent meta-analysis highlighted that *H. pylori*-positive patients who received probiotics combined with the eradication therapy present a significantly higher eradication rate as compared to the control group suggesting that *C. butyricum, B. licheniformis, Enterococcus + B. subtilis, L. acidophilus, Bifidobacterium + Lactobacillus + Enterococcus, S. boulardii*, and *Lactobacillus + Streptococcus lactis* are the most effective probiotics in eradication if added in 7-day triple therapy [98]. Contrariwise, the results of Lu et al. did not support these findings since the authors found no major benefit of probiotic supplementation on the eradication rate when compared to placebo [99]. Similarly, the meta-analysis of Dang et al. revealed no significant effect of probiotics on the eradication rate regardless if they were used alone or combined with standard antibiotic regimens [81]. Other studies also support the fact that probiotics prove no efficacy and safety in assisting with the eradication [100,101] (Table 2). Therefore, the results reported in the literature remain controversial and require further studies in order to clearly delineate the impact of probiotics on the *H. pylori* eradication rate. 

The evidence regarding the role of probiotics in children with *H. pylori* infection remains scarce. Nevertheless, a large study including 440 Thai children, among which 132 were infected with *H. pylori*, were divided into three groups: probiotics group—children who ate cheese with *L. gasseri LG21* strain for 12 months, placebo group—children who ate cheese without probiotics for 12 months, and control group—children who did not eat cheese, indicated an eradication rate of 29.3% in probiotics group, but the authors concluded that probiotics are not useful for preventing *H. pylori* infection or reinfection [102]. Another randomized double-blind placebo-control research which aimed to assess the efficacy of standard triple therapy involving amoxicillin, clarithromycin, and omeprazole in association with a fermented milk product supplemented with *L. casei DN-114001* strain in treating 86 children with *H. pylori* infection revealed that this combination increases the eradication therapeutic benefit in these children [103]. These findings were also supported by a randomized clinical trial including 65 children who received the same standard triple therapy but combined with 250 ml commercial yogurt supplemented with *Bifidobacterium animalis* [104]. Synbiotics, defined as an association between probiotics and non-digestible dietary compounds characterized by the ability to stimulate the growth of certain benefic bacteria for the host’s health called prebiotics [105], were also assessed in the eradication of pediatric *H. pylori* infection. Thus, Sirvan et al. proved that the addition of *B. lactis*-based synbiotics to the standard triple therapy using amoxicillin, clarithromycin, and lansoprazole significantly increases the eradication rate of *H. pylori* in children [106]. In addition, Feng et al. stated in a systematic review and network meta-analysis which compared probiotic-supplemented triple therapy with placebo that *L. casei* and multi-strain of *C. butyricum* and *B infantis* are the most effective probiotics for increasing *H. pylori* eradication rates in pediatric populations [107]. The same combination of multi-strain containing *C. butyricum* and *B. infantis* associated with 14-day standard triple therapy was found to be the most effective also in the study by Wen et al. performed on Asian children diagnosed with *H. pylori* infection [108] (Table 2). 

Antibiotics-associated side effects were proven to be significantly reduced when probiotics, i.e. *L. reuteri ATCC 55730* are administered in association with sequential therapy as was proved by a randomized double-blind placebo-controlled trial performed on 40 children with dyspeptic symptoms who were administered amoxicillin and omeprazole for the first 5 days, followed by omeprazole + clarithromycin + tinidazole [109]. A more recent network meta-analysis indicated that multi-strain of *L. rhamnosus* and *L. acidophilus* or *C. butyricum*, *B. mesentericus*, and *Streptococcus faecalis*, as well as a single strain of *S. boulardii* are the most effective in reducing the global incidence of standard triple therapy-associated side effects in children [107]. Another meta-analysis performed by Wen et al. on pediatric patients also revealed the multi-strain containing *C. butyricum* + *B. mesentericus* + *S. faecalis* to be the best in diminishing side effects when supplemented with 14-day triple therapy [108]. As for reducing the particular side effects, the authors found *B. infantis* + *B. bifidum + L. acidophilus* + *L. casei* + *L. reuteri* + *L. bulgaricus* + *Streptococcus* along with *L. acidophilus* + *B. bifidum* were the most beneficial for decreasing the incidence of vomiting, nausea, and diarrhea [108]. These findings are supported by a recent meta-analysis [110] which assessed 31 studies that reported taste disturbance, diarrhea, abdominal pain, nausea, vomiting, and constipation as the most common side effects. Thus, the authors proved that the incidence of these adverse events related to the eradication therapy was significantly lower in the probiotic group when compared to the control group. Other similar studies also indicated a significant reduction of standard eradication therapies-associated side effects such as antibiotics-related diarrhea in patients that were supplemented with probiotics [74,111,112,113] (Table 2). 

**Table 2:** The effects of probiotics on pediatric *H. pylori* infection.

Probiotics	Effect	Cons
*General*	eradication rate of *H. pylori* infection in 29.3% of children who use probiotics [102] no significant effect of probiotics on the eradication rate regardless if they were used alone or combined with antibiotics-standard regimens [81] probiotics prove no efficacy and safety in assisting with eradication [100,101]	probiotics are not useful for preventing *H. pylori* infection or reinfection [102] children with *H. pylori* infection have a lower amount of fecal *Bifidobacterium* spp. and a decreased *Bifidobacterium* to *E. coli* ratio in their feces as compared to *H. pylori*-negative children [90]
*L. reuteri +* sequential therapy in children	antibiotics-associated side effects are reduced → after *L. reuteri +* sequential therapy in children with dyspeptic symptoms [109]	–
*L. rhamnosus*, *L. acidophilus* or *C. butyricum*, *B. mesentericus*, and *Streptococcus faecalis*, or a single strain of *S. boulardii*	most effective in ↓ the therapy-associated side effects [107]	–
*C. butyricum* + *B. mesentericus* + *S. faecalis*	↓ side effects when supplemented with 14-day triple therapy [108]	–
*B. lactis* and *L. acidophilus*	supplementation with *B. lactis* and *L. acidophilus* → positive effect on *F. prausnitzii* growth + *H. pylori*-positive children present also ↓ IgA level, which is crucial in regulating gut microbiota [91].	children with *H. pylori* infection → fecal *F. prausnitzii* → ↓ gut inflammation triggered by *H. pylori* lipopolysaccharide [16].
Triple therapy (amoxicillin, clarithromycin, + omeprazole) + fermented milk product supplemented with *L. casei DN-114001*	↑ eradication therapeutic benefit in children with *H. pylori* infection [103]	–
Triple therapy + 250 ml yogurt supplemented with *Bifidobacterium animalis*	↑ eradication of *H. pylori* infection	–
*B. lactis*-based synbiotics + standard triple therapy (amoxicillin, clarithromycin + lansoprazole)	↑ the eradication rate of *H. pylori* in children [106].	–
Probiotic-supplemented triple therapy with *L. casei* and multi-strain of *C. butyricum* and *B infantis*	most effective probiotics for increasing *H. pylori* eradication rates in children [107]	–
*C. butyricum* and *B. infantis* + 14-day standard triple therapy	effective in children with *H. pylori* infection [108]	–
*B. infantis* + *B. bifidum + L. acidophilus* + *L. casei* + *L. reuteri* + *L. bulgaricus* + *Streptococcus* along with *L. acidophilus* + *B. bifidum*	most beneficial for ↓ vomiting, nausea, and diarrhea [108]	–

↓ decrease side effects. ↑ increase.

## 4. Conclusions

Despite the fact that *H. pylori* infection is commonly acquired during childhood, its life-threatening complications usually occur during adulthood suggesting that this is a long-term process that involves both *H. pylori* virulence factors and the host’s defense mechanisms. The eradication of this infection as early as possible represents a crucial need in pediatric patients. Several eradication regimens are available for the treatment of *H. pylori* infection, but multiple limitations are associated with each of them, especially in terms of antibiotic resistance and side effects. Although the use of probiotics alone in the eradication of this infection is not generally supported, their association with the standard antibiotic-based regimens might improve the eradication rates and decrease the most important side effects induced by antibiotics. Nevertheless, the evidence of their role in pediatric patients remains scarce, and further studies in this age group would definitely improve the knowledge regarding their short- and long-term positive and negative effects. 
